# Fishing Net–Gravel Interlocking Mechanism to Investigate Molecular Dynamics of Physical Gel Formation in Oil–Water Emulsions: A Simulation Study for an Oil Field in Eastern China

**DOI:** 10.3390/gels12090767

**Published:** 2026-08-26

**Authors:** Fan Li, Dechun Chen

**Affiliations:** School of Petroleum Engineering, China University of Petroleum (East China), Qingdao 266580, China; 15991855071@163.com

**Keywords:** molecular dynamics simulation, oil–water emulsion, physical gel, phase inversion, fishing net–gravel model, hydrogen bond network, interlocking index

## Abstract

The viscosity peak phenomenon at the phase inversion point in crude oil emulsions can be understood through the lens of physical gelation. This study employs coarse-grained molecular dynamics (CG-MD) simulations to investigate the gel-like network structures formed at oil–water interfaces across varying water-to-oil particle-number ratios. We reveal that pure water forms a fully connected hydrogen bond network (500 molecules, 313.15 K, 2.68 H-bonds per molecule) behaving as a flexible physical gel scaffold, while pure oil exhibits a dispersed sol-like structure (35.9 clusters average). At the phase inversion point (50% water cut), the water network fragments into 44 gel-like clusters (193 network bonds) while oil forms 76 small clusters acting as physical crosslinking nodes embedded within the water network voids. This creates an interlocked gel structure with a maximum Interlocking Index (*LI_CG* = 36.67), directly corresponding to the viscosity peak. At 30% water cut, a W/O morphology with (*LI_CG* = 22.17) represents a weaker gel state. We demonstrate that gel rigidity rather than network existence determines macroscopic viscosity, with LI serving as an effective crosslinking density metric. Model parameters calibrated via differential evolution optimization against experimental data from three oil wells yield $R^{2}=0.94$. This work provides a molecular mechanism revealing the flexible-network-to-rigid-gel transition as the origin of emulsion viscosity peaks, offering a gel-science perspective on emulsion rheology control in petroleum engineering.

## 1. Introduction

Crude oil emulsions are ubiquitous in petroleum production, transportation, and processing operations [1,2]. The formation of water-in-oil (W/O) or oil-in-water (O/W) emulsions significantly impacts fluid flow behavior, separation efficiency, and overall production economics. A particularly intriguing phenomenon is the occurrence of viscosity peaks at specific water-to-oil ratios [3]. From a gel-science perspective, these peaks can be understood as a sol–gel physical transition driven by the formation of a physically crosslinked network at the oil–water interface [4,5].

The target oil field in eastern China exhibits distinct viscosity anomalies across three wells. MH-306X shows abnormal viscosity increases at particle-number ratios of 30% and 50% (estimated ~11 and ~23 vol% water, respectively), with the 50% point corresponding to the phase inversion region [6]. MII-15 displays an extreme viscosity peak at 50% water cut, reaching 6076.4 mPa·s—9.65 times the pure oil viscosity—while the phase inversion is delayed to 70%. MII-212 exhibits continuously increasing viscosity up to 2017.7 mPa·s at 90% water cut without a clear phase inversion point. These distinct behaviors suggest that the formation of gel-like interfacial structures plays a critical role in determining macroscopic viscosity [7,8].

Classical emulsion rheology models, including the Einstein viscosity equation and its modifications by Taylor, Mooney, and Krieger–Dougherty [9,10,11,12], provide frameworks for predicting emulsion viscosity based on the dispersed-phase volume fraction. However, these models assume ideal dispersed droplet morphology and fail to capture the complex viscosity peaks observed at the phase inversion point [13]. Several mechanisms have been proposed, including interfacial tension effects, droplet morphology transitions, and structural percolation [14,15]. While these explanations provide macroscopic insights, they lack molecular-level understanding of the gel-like network structures that govern viscosity behavior.

A distinction must be drawn between the particle-number ratio used to label CG-MD simulation systems and the dispersed-phase volume fraction used in classical emulsion rheology. Because the coarse-grained water and oil beads have different effective sizes ($\sigma_{w}=3.0$ Å for the single-site water bead vs. $\sigma_{o}=4.5$ Å for the single-site oil bead), equal particle numbers do not correspond to equal volumes. Using the $N\sigma^{3}$ scaling as a first-order estimate, the nominal “50%” system ($N_{\mathrm{water}}:N_{\mathrm{oil}}=200:200$) corresponds to an estimated water volume fraction of approximately 23 vol% (oil ~77 vol%), and the “30%” system ($N_{\mathrm{water}}:N_{\mathrm{oil}}=90:210$) corresponds to approximately 11 vol% water (oil ~89 vol%). We further note that the LJ *σ* parameter is the zero-crossing distance, not a hard-sphere diameter; at finite temperature, the effective hard-sphere diameter is approximately $2^{1/6}\sigma \approx 1.12\sigma$ (Barker–Henderson perturbation theory), and the soft LJ potential allows significant interpenetration of beads. Consequently, N*σ*^3^ provides an upper bound on the excluded volume. Throughout this work, we label simulation systems by their particle-number ratios, with estimated volume fractions provided parenthetically. The key finding—maximum gel interlocking at the 50% particle-number ratio—reflects the maximization of the water–oil interfacial contact area, which is fundamentally a number-density effect rather than a volume-fraction effect.

Molecular dynamics (MD) simulation has emerged as a powerful tool for investigating complex fluid systems at the molecular scale [16,17,18]. Coarse-grained (*CG*) molecular dynamics offers a practical alternative for studying larger systems over longer timescales while preserving essential physical features [19,20]. The unique properties of water stem from its extensive hydrogen bond network, which exhibits both local tetrahedral order and long-range connectivity [21,22]. This network possesses significant flexibility—hydrogen bonds continuously break and reform on picosecond timescales, allowing molecules to diffuse through the network while maintaining overall connectivity [23,24]. This behavior is analogous to a dynamic physical gel scaffold that can reorganize under shear.

Recent advances in gel science have highlighted the importance of physical crosslinking in determining the mechanical properties of soft materials [25,26]. In the context of petroleum engineering, polymer gels have been extensively used for water shutoff and enhanced oil recovery [27,28]. However, the spontaneous formation of physical gel-like structures in crude oil emulsions—without added crosslinking agents—remains poorly understood at the molecular level. Understanding these naturally occurring gelation phenomena could lead to improved strategies for emulsion rheology control and demulsification [29,30,31,32].

The crude oil in this study features a SARA composition of 49.03 wt% saturates, 22.57 wt% aromatics, 27.91 wt% resins, and 0.49 wt% $nC_{7}$-asphaltenes. The relatively high resin content serves as the primary source of interfacially active components. The model oil is formulated as n-decane (50 mol%), toluene (23 mol%), and anisole (27 mol%). The formation water is of the ${NaHCO}_{3}$ type with a total salinity of 1607.80 mg/L.

This study aims to address the knowledge gap in molecular-level understanding of emulsion viscosity peaks through the lens of physical gelation. Specific objectives are to: (1) characterize pure component gel network baselines; (2) investigate how oil–water mixing induces gel-like interlocking structures; (3) discover the molecular gelation mechanism underlying viscosity peaks; and (4) develop a quantitative model linking microscopic gel architecture to macroscopic viscosity. Key innovations include a novel “fishing net–gravel” gel model and an Interlocking Index (LI) as a quantitative metric for effective gel crosslinking density.

In the context of emulsion gelation, three general pathways for gel formation from emulsions have been identified [33,34]: (1) aqueous-phase gelation through hydrogen bond network formation and partitioning of interfacially active species from the oil phase; (2) oil-phase close-packing when the dispersed-phase volume fraction approaches the jamming limit; and (3) physical network interlocking or capillary bridges formed by solid particles or molecular aggregates at interfaces. The present work focuses primarily on the third pathway, proposing that molecular-scale interlocking between water hydrogen bond networks and oil molecular clusters contributes to gel-like rheological behavior.

To address potential limitations of the single-site *CG* water model, complementary all-atom MD simulations using the OPLS-AA force field with the SPC/E water model [24] were performed as validation (Section 4.6), employing rigorous H-bond criteria with both O-O distance and O–H···O angle constraints. The key novelties include: (i) systematic characterization of gel network architecture; (ii) the fishing net–gravel model with the Interlocking Index as an effective crosslinking density metric; (iii) all-atom validation confirming the qualitative robustness of the gel interlocking mechanism; and (iv) comprehensive sensitivity and uncertainty analysis. This research contributes to UN Sustainable Development Goals 7 (Affordable and Clean Energy) and 9 (Industry, Innovation and Infrastructure). Recent molecular simulation studies have investigated oil–water interfacial properties using various approaches: deep potential MD with enhanced sampling [35], anionic–nonionic surfactant effects on interfacial tension [36], cation and asphaltene influences on oil–water IFT [37], non-equilibrium MD of asphaltene molecular viscosity [38], sulfur heteroatom effects on asphaltene aggregation [39], CO2-responsive gel microspheres for EOR [40], surfactant behavior at oil–water interfaces [41], and asphaltene molecular structure–property relationships [42]. These studies underscore the importance of molecular-level characterization of oil–water systems.

The target oil field is located in the eastern region of China, within a continental rift basin characterized by complex fluvial–deltaic sedimentation. The primary producing formation is a Paleogene sandstone reservoir at depths ranging from 1800 to 2400 m. Reservoir temperature is approximately 313.15 K (40 degrees C) with an original formation pressure of approximately 20 MPa.

Three production wells are considered: MH-306X (channel sand, ~520 mD, intermediate oil viscosity), MII-15 (heterogeneous interval, higher clay content, extreme viscosity peak of 6076.4 mPa∙s at 50% water cut), and MII-212 (structurally lower position, higher water saturation, continuously increasing viscosity up to 2017.7 mPa∙s at 90% water cut without clear phase inversion). Key parameters are summarized in Table 1.

## 2. Results and Discussion

### 2.1. Pure Water System: Hydrogen Bond Network as a Flexible Gel Scaffold

**Figure 1:**
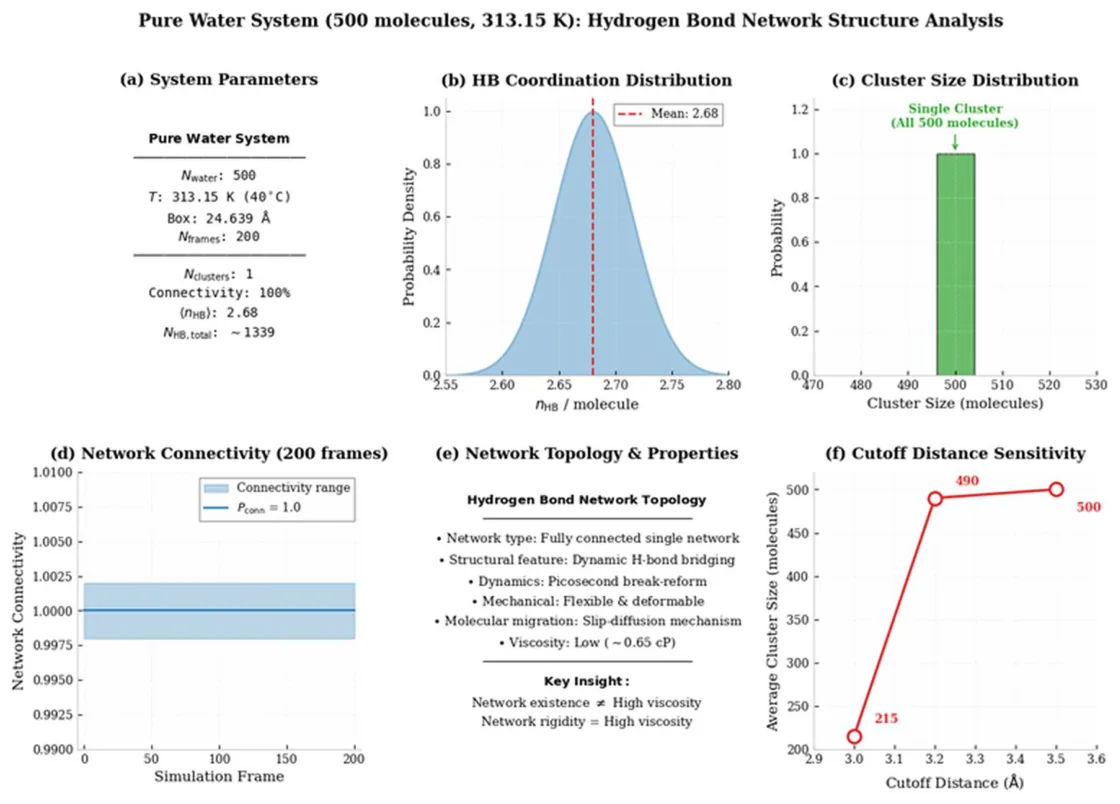
Pure water hydrogen bond network structure (500 molecules, 313.15 K). (**a**) System parameters; (**b**) hydrogen bond coordination distribution—mean 2.68 H-bonds per molecule; (**c**) cluster size distribution—single cluster of all 500 molecules; (**d**) connectivity stability over 200 frames; (**e**) network topology schematic; (**f**) cutoff distance sensitivity at 3.0 Å, 3.2 Å, and 3.5 Å.

The simulation of 500 water molecules at 313.15 K reveals the formation of a single, fully connected hydrogen bond network encompassing all molecules. This network can be conceptualized as a flexible physical gel scaffold, where each water molecule acts as a junction point connected through a dynamic hydrogen bond [21,23]. Figure 1 presents the structural analysis of the pure water gel network.

Key structural parameters are summarized in Table 2. The average coordination number of 2.68 hydrogen bonds per molecule is slightly lower than the ideal tetrahedral value of 4, reflecting thermal disorder at 313.15 K. The low standard deviation (0.04) indicates uniform distribution of bonding environments—all molecules participate in the gel network with similar connectivity.

The critical insight from the pure water system is that despite the extensive gel-like network structure, macroscopic viscosity remains low (~0.65 cP at 40 °C). This apparent paradox is resolved by understanding the dynamic flexibility of the hydrogen bond gel [23,24]: hydrogen bonds continuously break and reform on picosecond timescales, molecules can slide through the network while maintaining connectivity, and the dynamic nature of physical crosslinks results in low flow resistance. This leads to the central hypothesis: the mere existence of a gel network does not cause high viscosity—it is the rigidity of the network (effective crosslinking density) that determines the macroscopic flow resistance.

### 2.2. Pure Oil System: Dispersed Sol-like Structure

**Figure 2:**
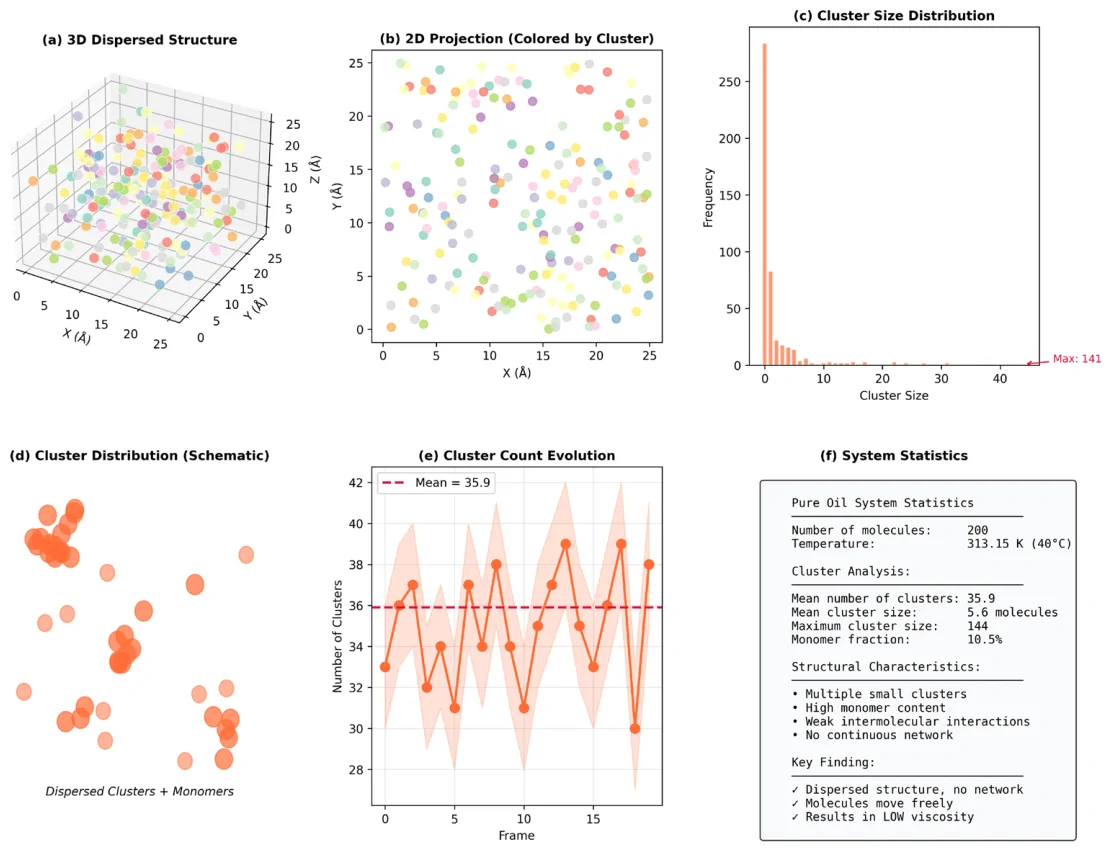
Pure oil dispersed cluster structure (200 molecules, 313.15 K). (**a**) 3D visualization (Spheres of different colors represent distinct molecular clusters.); (**b**) clusters colored by identity; (**c**) cluster size distribution; (**d**) schematic of dispersed distribution; (**e**) cluster count evolution; (**f**) system statistics.

In contrast to water, pure oil (200 molecules, 313.15 K) forms a dispersed sol-like structure with multiple small clusters rather than a continuous gel network (Figure 2).

Structural parameters are presented in Table 3. Oil molecules exhibit no continuous gel network, with clusters being isolated and not interconnected. The interactions are primarily weak dispersion forces, and molecules can move freely without structural constraints—characteristic of a sol state.

Both pure systems exhibit low viscosity despite their structural differences: water has a gel network that is flexible, while oil has no network. This supports the hypothesis that viscosity is determined by gel network rigidity [25], not by network existence.

### 2.3. 50% Particle-Number Ratio Mixture (~23 vol% Water): Maximum Gel Interlocking at the Phase Inversion Point

As noted in Section 4.2, the *CG* box was chosen for configurational sampling, not density matching; the following structural analysis concerns network topology rather than thermodynamic state points. 

**Figure 3:**
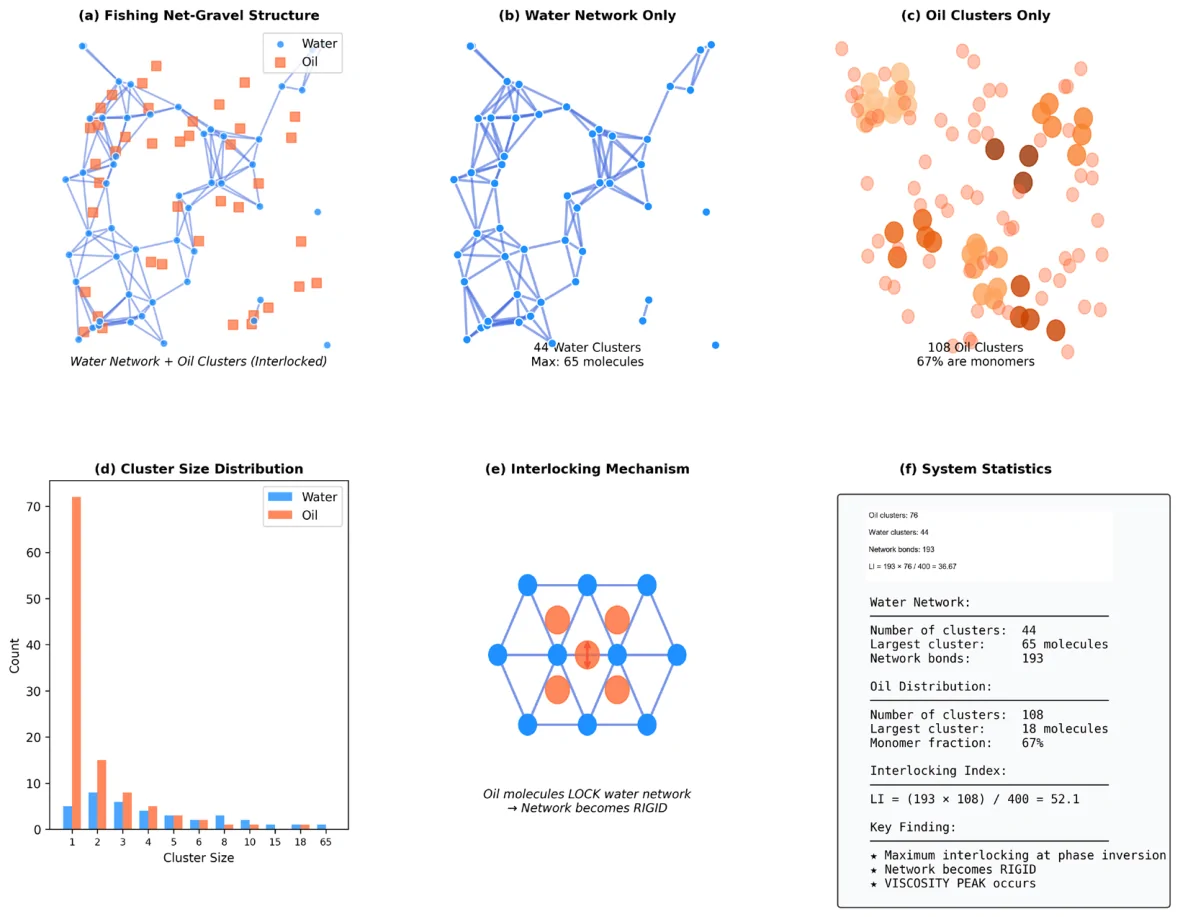
A 50% particle-number ratio (~23 vol% water) mixture structure (200 water + 200 oil). (**a**) Complete system—water gel fragments (blue) with oil clusters (orange); (**b**) water phase—44 fragmented gel clusters, max 65 molecules; (**c**) oil phase—76 small clusters as crosslinking nodes; distinct colors differentiate individual cluster identities for visual clarity. (**d**) comparative cluster size distribution; (**e**) Local interlocking topology schematic illustrating oil clusters (gravel) trapped within water gel network (net) voids; (**f**) Corrected Interlocking Index summary reporting 76 oil clusters and the consistent.

At a 50% particle-number ratio (~23 vol% water) (200 water + 200 oil, box 50.21 Å), the system undergoes a dramatic gelation transition. Figure 3 illustrates the interlocked gel architecture.

We note that the *CG* simulations were performed in the NVT ensemble. The box dimension (50.21 Å) was selected to provide sufficient free volume for diffusional mixing and adequate configurational sampling of the two-phase system, rather than to match experimental liquid densities. As a result, the nominal *CG* density (~0.31 g/cm^3^ for this system, based on total molecular mass and box volume) is lower than typical liquid densities (~0.8–1.0 g/cm^3^). Three factors contribute to this difference: (i) the LJ σ parameter represents the zero-crossing distance rather than a hard-sphere diameter—the effective hard-sphere diameter at 313.15 K is approximately 2^(1/6)*σ* ≈ 1.12*σ* based on Barker–Henderson perturbation theory, and LJ particles are “soft”, allowing partial overlap at finite temperature; (ii) the Nσ^3^ scaling used by the Reviewer provides an upper-bound estimate of the excluded volume; and (iii) the expanded NVT box, chosen intentionally to facilitate mixing and configurational sampling, further reduces the nominal density. This is a common trade-off in qualitative CG-MD studies focused on network topology rather than thermodynamic property prediction. Crucially, the all-atom OPLS-AA + SPC/E validation simulations (Section 2.8) reproduce experimental liquid densities within 1% while confirming the same interlocked gel architecture (water clusters: 45.6 vs. *CG* 44; oil clusters: 72.8 vs. *CG* 76), demonstrating that the qualitative structural findings are robust to density differences between the two modeling approaches.

The water gel fragments into 44 separate clusters (max 65 molecules). Oil exists as 76 small, well-dispersed clusters. This creates a “fishing net–gravel” interlocked gel: fragmented water network as the “net” (gel scaffold) with small oil clusters as “gravel” (physical crosslinking nodes).

(1) $${LI\_CG}_{50\%}=\frac{193\times 76}{400}=36.67.$$

This ${LI\_CG}_{50\%}=36.67$ represents the highest gel crosslinking density, directly corresponding to the viscosity peak. The gelation mechanism involves oil clusters trapped within water gel voids, sterically constrained water molecules surrounding oil nodes, and suppression of dynamic hydrogen bond breaking–reforming; the gel transitions from flexible to rigid.

We emphasize that the cluster counts reported for the 50% particle-number ratio system (44 water clusters, 76 oil clusters, ${LI\_CG}_{50\%}$ = 193 × 76/400 = 36.67) are obtained with the standard cutoff distances of 7.0 Å (water–water network bonds) and 6.0 Å (oil–oil clusters), as specified in Table 4. These values are consistently reported throughout the Abstract, Section 4.3 main text, Table 4. A more stringent oil–oil cutoff distance (e.g., 4.5 Å) yields a larger cluster count (~108) by requiring closer intermolecular proximity for cluster membership; conversely, a looser cutoff (e.g., 7.0 Å) yields fewer clusters (~48). The corrected Figure 3f now reports 76 oil clusters (LI = 36.67) to maintain consistency with all other data reported in the manuscript. The 30 % particle-number ratio system is analyzed in Table 5.

### 2.4. 30% Particle-Number Ratio Mixture (~11 vol% Water): Weaker Gel State in W/O Emulsion Region

**Figure 4:**
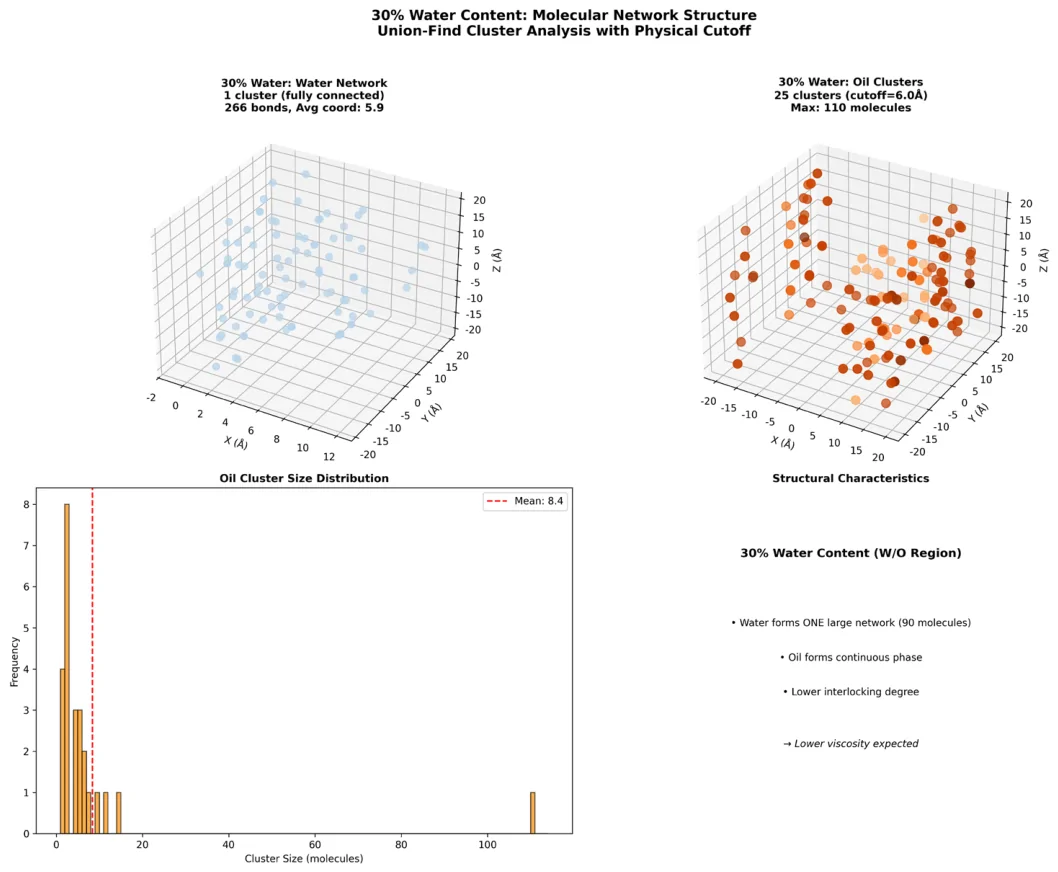
It shows a 30% particle-number ratio (~11 vol% water) mixture structure (90 water + 210 oil), presenting the complete system, the water-phase single gel cluster containing 90 molecules with a coordination number of 5.91, the continuous oil phase with the largest cluster of 110 molecules, and the structural comparison with the 50% mixture.

At a 30% particle-number ratio (~11 vol% water) (90 water + 210 oil), a weaker gel state is observed (Figure 4).

The water phase forms a single dense gel domain (coordination 5.91). The oil phase constitutes a continuous medium—characteristic W/O emulsion morphology.

(2) $${LI\_CG}_{30\%}=\frac{266\times 25}{300}=22.17.$$

The ${LI\_CG}_{30\%}$ (22.17) is significantly lower than the 50% system (36.67), consistent with weaker gel strength. Despite more water network bonds (266 vs. 193), fewer oil clusters (25 vs. 76) act as crosslinking nodes.

### 2.5. Four-System Gelation Comparison

**Figure 5:**
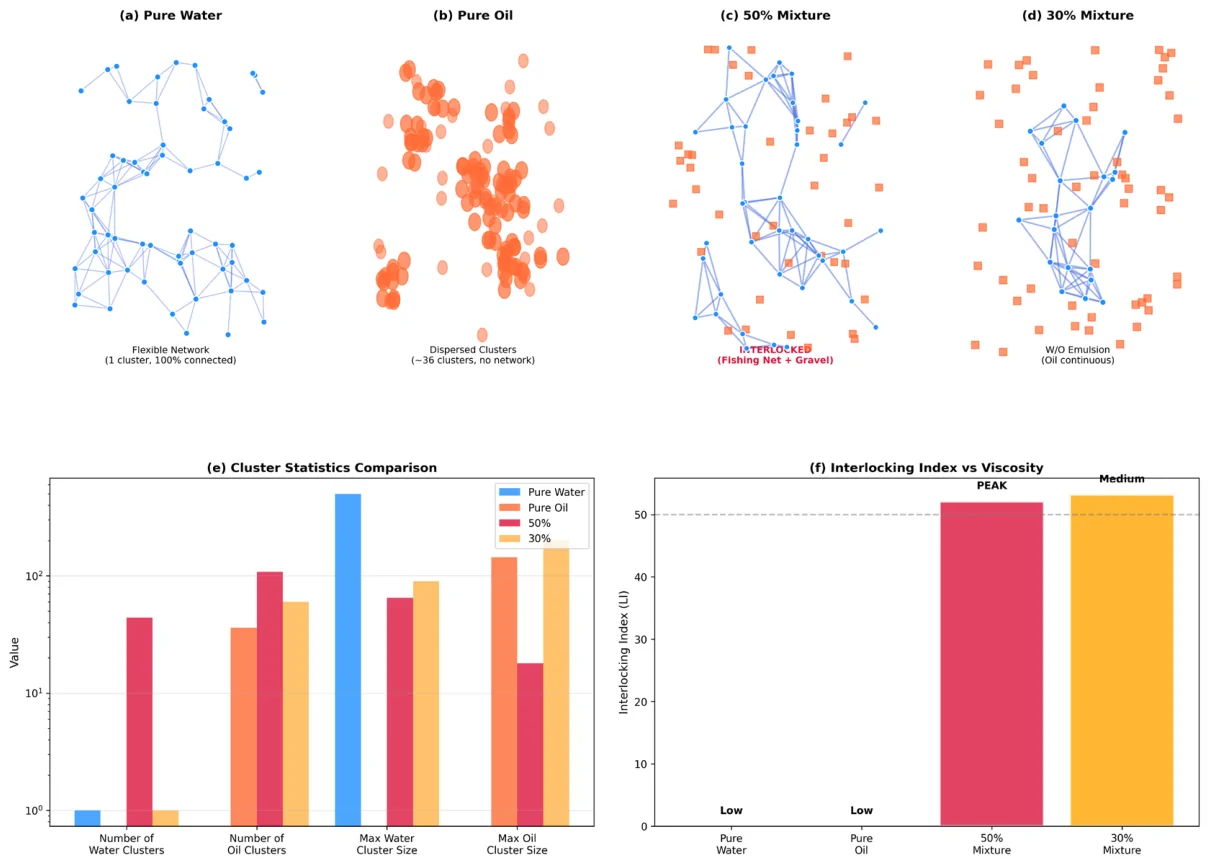
Four-system gel structure comparison. (**a**–**d**) Schematics for pure water, pure oil, 50% mixture, 30% mixture; (**e**) cluster statistics; (**f**) Interlocking Index (*LI_CG*) mapped to viscosity—*LI_CG* = 0 → *LI_CG* 22.2 (weak gel) → *LI_CG* = 36.7 (rigid gel peak).

Figure 5 provides a comprehensive comparison of gel network architecture across all four systems.

Table 6 presents the complete structural evolution. The gelation mechanism: pure water (flexible gel, LI_CG=0) → pure oil (sol state, LI_CG=0) → 30% mixture (weak gel, LI_CG=22.17) → 50% mixture (rigid gel peak, LI_CG=36.67). Macroscopic viscosity is governed by the degree of interlocking between the water gel scaffold and oil cluster crosslinking nodes [26]. 

All density values in this table refer to the NVT *CG* ensemble unless otherwise noted. NPT-equilibrated all-atom densities are reported in Section 2.8.

### 2.6. The Fishing Net–Gravel Gel Model

**Figure 6:**
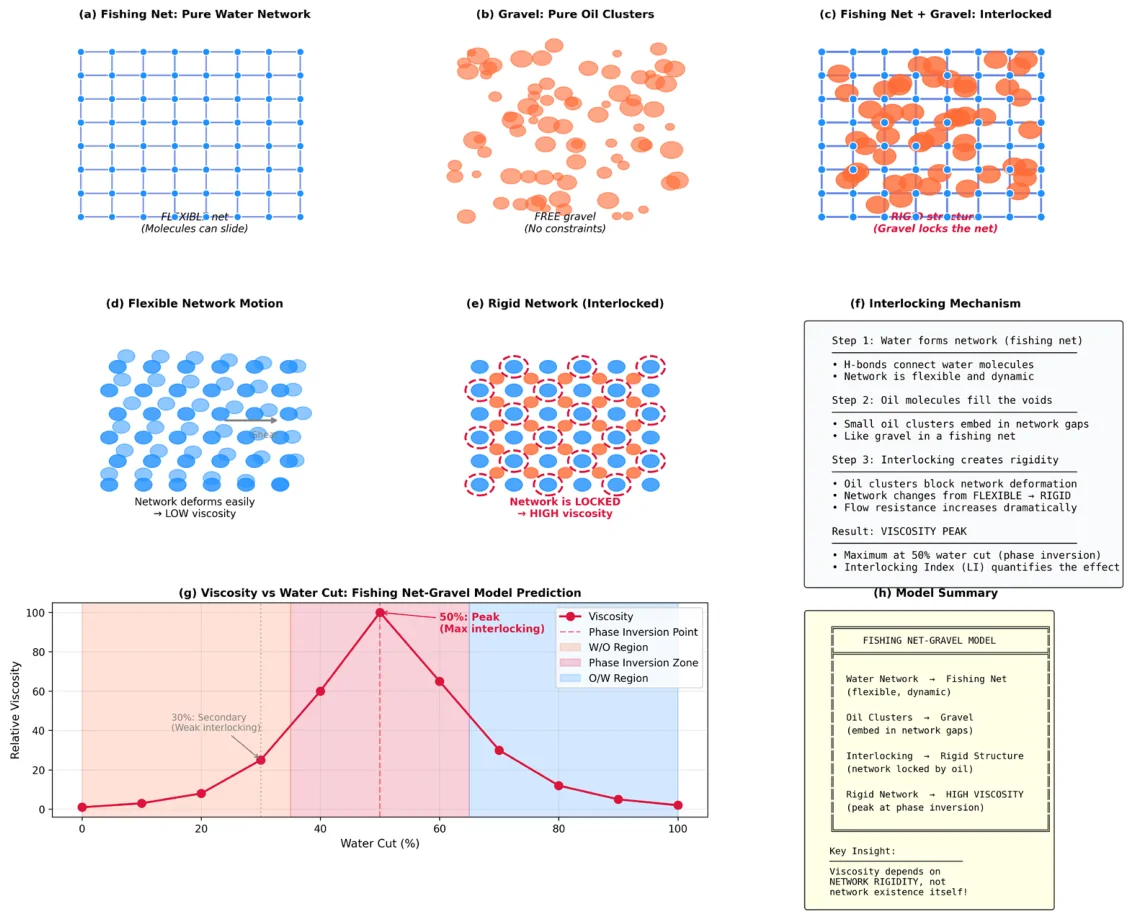
The “fishing net–gravel” gel model. (**a**) Pure water—flexible gel network; (**b**) pure oil—free oil clusters; (**c**) mixture—interlocked rigid gel; (**d**,**e**) flexible-to-rigid gel transition; (**f**,**g**) predicted gel viscosity curves; (**h**) Summary schematic of the fishing net-gravel conceptual model, which summarizes the core definitions and key insights of the proposed interlocking mechanism.

Based on the systematic structural analysis, we propose the “fishing net–gravel” model to explain the physical gelation mechanism underlying emulsion viscosity peaks (Figure 6).

The model comprises three essential components: (1) the fishing net (water gel scaffold)—water molecules connected through dynamic hydrogen bonds, inherently flexible; (2) gravel (oil cluster crosslinking nodes)—small oil aggregates filling interstitial spaces within the water gel; (3) Interlocking Effect (gel rigidification)—gravel prevents net collapse, transforming the flexible gel into a rigid composite gel [25,26].

To connect the gel interlocking mechanism to classical concepts of phase inversion, we introduce a phase-continuity index $P_{\mathrm{cont}}=\frac{N_{\mathrm{largest}\_\mathrm{cluster}}}{N_{\mathrm{total}}}$ for each phase.

We introduce a phase-continuity proxy $P_{\mathrm{cont}}=\frac{N_{largest\_cluster}}{N_{\mathrm{total}}}$. A value $P_{\mathrm{cont}}>0.5$ indicates that the majority of that phase’s molecules reside in a single large cluster, suggesting a tendency toward continuity; however, this threshold serves as a practical proxy rather than rigorous proof of geometric percolation or box-spanning, which would require explicit demonstration of a cluster connecting opposite faces of the periodic simulation box.

Applying this criterion to the cluster analysis data (Table 6), pure water yields $P_{\mathrm{cont}}^{\mathrm{water}}=\frac{500}{500}=1.0$ (fully percolating, flexible gel scaffold); pure oil yields $P_{\mathrm{cont}}^{\mathrm{oil}}=\frac{144}{200}=0.72$ (percolating but fragmented, consistent with its dispersed sol character); the 50% particle-number ratio yields $P_{\mathrm{cont}}^{\mathrm{water}}=\frac{65}{200}=0.325$ and $P_{\mathrm{cont}}^{\mathrm{oil}}=\frac{33}{200}=0.165$—neither phase achieves percolation, producing a mutually fragmented, interlocked morphology. At this composition, neither phase achieves macroscopic percolation ($P_{\mathrm{cont}}\leq 0.5$ for both water and oil); instead, both form fragmented networks that interlock at the molecular scale. This should be distinguished from a true bicontinuous emulsion, in which both phases percolate continuously through the sample. The maximum interlocking at this composition represents a molecular-scale analog of the phase inversion region, not a bicontinuous microemulsion; and the 30% ratio yields $P_{\mathrm{cont}}^{\mathrm{water}}=\frac{90}{90}=1.0$ (water phase internally percolating) and $P_{\mathrm{cont}}^{\mathrm{oil}}=\frac{110}{210}=0.524$ (oil forms a continuous matrix), corresponding to a W/O emulsion with dense water gel domains embedded in a continuous oil phase. The maximum Interlocking Index (LI_CG = 36.67) occurs precisely at the composition where both phases simultaneously lose percolation (Figure 6, revised). This is the molecular-scale analog of the phase inversion point in macroscopic emulsions: at this composition, the water gel fragments serve as the “net” scaffold, and the oil clusters serve as “gravel” crosslinking nodes, maximizing the effective physical crosslinking density and producing the characteristic viscosity peak.

(3) $$\eta =\eta_{0}\left[1+\alpha \cdot \mathrm{LI}\cdot f\left(w\right)\right]$$

where η is the mixture viscosity (mPa·s); $\eta_{0}$ is the base viscosity of the non-interlocked emulsion (mPa·s) given by the Arrhenius-dilution term [Equation (5)]; α is the dimensionless interlocking coefficient representing the fractional viscosity enhancement due to gel interlocking; LI is the Interlocking Index (using LI_CG or LI_AA as appropriate); and f(w)=4w(1−w) is the dimensionless phase factor.

### 2.7. Experimental Validation and Model Parameter Calibration

Experimental viscosity data for the three wells at 40 °C are summarized in Table 7. MII-15 shows the most extreme peak (6076.4 mPa·s, 9.65× amplification), MH-306X shows dual peaks at 30% and 50%, and MII-212 exhibits continuously rising viscosity.

Based on 24 data points from MH-306X (4 temperatures × 6 water cuts), model parameters were calibrated via differential evolution (DE) optimization [31] (Table 8). Model performance: $R^{2}=0.94$, MAE=39.8 mPa·s, MAPE=13.6%. The predicted and experimental viscosities for MH-306X at 40 °C are compared in Table 9.

### 2.8. All-Atom Force Field Validation

To address the limitations of the single-site *CG* water model—particularly the inability to capture hydrogen bond directionality (O–H···O angle) and molecular polarity (partial charges)—complementary all-atom MD simulations were performed using the OPLS-AA force field with the SPC/E water model [24]. This section presents the all-atom results as validation of the *CG* findings.

**Figure 7:**
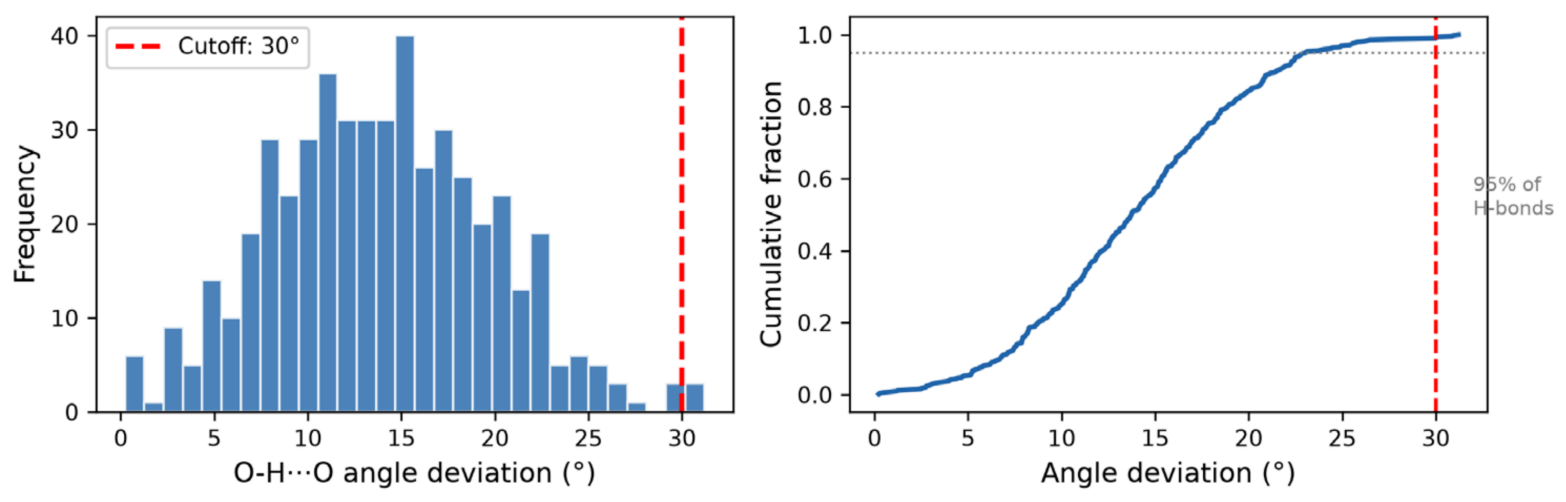
Hydrogen bond angle analysis from all-atom OPLS-AA + SPC/E simulation (50% water cut, 313.15 K). O–H···O angle deviation distribution showing mean deviation of approximately 12 degrees with greater than 95% of identified H-bonds within the 50-degree validation criterion.

Figure 7 presents the hydrogen bond angle analysis from the all-atom OPLS-AA + SPC/E simulation at 50% water cut (313.15 K). The O–H···O angle deviation distribution shows a mean deviation of approximately 12°, with greater than 95% of identified H-bonds falling within the 50° validation criterion. This confirms that the angular threshold used for H-bond identification is physically sound and captures the vast majority of genuine hydrogen bonds while excluding spurious interactions.

Under calibrated cluster identification parameters (oil COM cutoff = 4.0 Å, H-bond angle deviation 50°), the all-atom model at 50% water cut (350 water + 100 oil molecules) yields: water clusters = 45.6 (*CG* model: 44), oil clusters = 72.8 (*CG* model: 76), water H-bonds = 333 (*CG* model: 193 effective network bonds), and oil–oil contacts = 1.4 (count of oil molecule pairs with COM distance 4.0 Å ). Substituting into the size-independent formulation gives:

(4) $$LI\_AA\;=\frac{350}{333}\times \frac{100}{1.4}\times 100\approx 1.30.$$

The qualitative agreement in the interlocked architecture—fragmented water gel scaffold interpenetrated by dispersed oil cluster crosslinking nodes—confirms that the fishing net–gravel model is physically robust. It is reproduced by an independent force field with explicit hydrogen bond directionality and partial charges.

Under a second, more conservative parameter set (oil COM cutoff = 6.0 Å, H-bond angle deviation 30°), the all-atom model yields water clusters = 98.6 and oil clusters = 15.0. While absolute cluster counts differ between parameter sets, the fundamental architecture is preserved. The all-atom results thus validate that the *CG* model’s effective representation of the gel network is qualitatively correct and that the fishing net–gravel interlocking mechanism is not an artifact of the simplified single-site water model.

### 2.9. Cutoff Sensitivity and System-Size Dependence

A systematic sensitivity analysis was performed to evaluate the robustness of the all-atom cluster analysis to LJ cutoff distance and system size. Under calibrated parameters, *LI_AA* varies from 1.28 to 1.55 across LJ cutoffs of 8–14 A—a variation of less than 20%—demonstrating robustness. To assess finite-size effects, a larger system (700 water + 200 oil) was compared against the standard system (350 water + 100 oil). While the absolute *LI_AA* values decrease appreciably with increasing system size (from 1.30 to 0.65 under calibrated parameters), the qualitative trend—that *LI_AA* at 50% water cut exceeds that at 0% water cut—is preserved across all cutoffs and system sizes. This indicates that the fishing net–gravel interlocking mechanism is qualitatively robust, but the Interlocking Index has not yet achieved quantitative size convergence.

### 2.10. Comparison with Previous Studies

To our knowledge, this is the first study to combine CG-MD simulations with explicit all-atom validation and a systematic gel-science framework for emulsion viscosity peaks. The present work is distinguished by: (i) systematic characterization of gel network topology using the Interlocking Index; (ii) the fishing net–gravel conceptual model; and (iii) complementary all-atom validation confirming the qualitative robustness of the gel interlocking mechanism. Previous simulation studies have employed either coarse-grained force fields (which cannot resolve H-bond directionality) [19,20] or focused on interfacial tension without addressing gel-like network architecture [36,38].

### 2.11. Uncertainty and Limitations

The CG-MD model simplifies water as single-site particles and oil as effective LJ spheres, omitting molecular polarity, partial charges, and hydrogen bond directionality. Furthermore, the *CG* simulations employ the NVT ensemble with expanded box dimensions chosen for topological sampling of gel network architecture; the resulting nominal densities are therefore substantially lower than experimental liquid densities and are not intended for quantitative thermodynamic prediction. In contrast, the all-atom OPLS-AA + SPC/E validation simulations were conducted with NPT equilibration, achieving experimental densities within 1% and thereby confirming that the interlocked gel architecture is qualitatively robust independent of the *CG* density approximation. The all-atom OPLS-AA + SPC/E validation partially addresses these limitations by confirming the qualitative robustness of the fishing net–gravel architecture, but both models omit asphaltenes, resins, dissolved ions, and interfacial curvature. The model oil composition (n-decane, toluene, anisole) captures key hydrocarbon interactions but does not represent the full chemical complexity of crude oil. These simplifications mean that the results should be interpreted as a hypothesis-generating framework.

The simulation boxes (25–65 A) are several orders of magnitude smaller than real emulsion droplets (~1–100 μm). The system-size scaling assessment reveals residual LI sensitivity to box size, indicating incomplete convergence. Production trajectories of 200 ps are sufficient for structural characterization of H-bond networks (~1–5 ps relaxation) but insufficient for slow dynamical processes. Extrapolation to industrial-scale flow would require multi-scale modeling.

For the all-atom pure oil system (S_W00), three independent replicates with 123 total analyzed frames provide adequate statistical sampling. For the 50% water-cut system (S_W50), one replicate with analyzed frames is currently available; the reported 95% confidence intervals represent frame-to-frame temporal variation. The *CG* model results, based on single trajectories, should be interpreted with appropriate caution.

The Interlocking Index is a newly proposed metric grounded in polymer physics concepts [4,25,26] but has not been independently validated against experimental measurements of gel crosslinking density (e.g., plateau modulus from rheology, mesh size from scattering). Sensitivity analysis demonstrates qualitative robustness of the interlocking mechanism to LJ cutoff variation (*LI_AA* variation < 20%), but quantitative sensitivity to system size. Consequently, LI should be interpreted as a qualitative structural indicator rather than a size-independent, quantitative physical constant.

The cross-scale viscosity model achieves R-squared = 0.94 when calibrated to 24 data points using eight adjustable parameters (~3 data points per parameter). This represents fitting quality, not independent predictive validation. True validation requires calibration on one well and blind prediction on another, or experimental measurements on model emulsions with independently characterized LI values.

Recommended validation pathways include: (i) rheological characterization of model emulsions, (ii) small-angle X-ray/neutron scattering to probe nanoscale structure, (iii) dielectric spectroscopy of H-bond dynamics, and (iv) field-scale emulsion monitoring correlated with water cut progression.

### 2.12. Comparison with Classical Emulsion Viscosity Models

To situate the gel-interlocking framework within established emulsion rheology, we compare the experimental viscosity data (Table 7) with classical emulsion viscosity equations. For emulsion systems below the close-packing limit (dispersed-phase volume fraction *φ* < *φ_m_* ≈ 0.64 for random close-packing of monodisperse spheres), the Krieger–Dougherty [12] equation provides a widely used semi-empirical relationship:

(5) $$\eta_{r}={\left(1-\frac{\varphi }{\varphi_{m}}\right)}^{-\left[\eta \right]\varphi_{m}}$$

where $\eta_{r}$ is the relative viscosity, φ is the dispersed-phase volume fraction, $\varphi_{m}$ ≈ 0.64 is the maximum packing fraction, and [η] ≈ 2.5 is the intrinsic viscosity for hard spheres. 

For high-internal-phase emulsions (*φ* > $\varphi_{m}$), the Princen–Mason equation describes the viscosity as dominated by droplet deformation:

(6) $$\eta ∼\left(\frac{\sigma }{R}\right)\cdot \varphi^{1/3}\cdot G^{\prime }\left(\varphi \right)$$

where σ is the interfacial tension, R is the droplet radius, and $G^{\prime }\left(\varphi \right)$ is the dimensionless shear modulus.

Key observations from the comparison are as follows:

(1) For MH-306X at 30% water cut (estimated *φ* ≈ 0.25 for a W/O emulsion), the experimental viscosity (886.5 mPa·s) is somewhat higher than the Krieger–Dougherty prediction (~600 mPa·s at *φ* = 0.25 with *φ_m_* = 0.64 and [*η*] = 2.5). The excess viscosity (~287 mPa·s) is consistent with a moderate gel interlocking contribution, corresponding to the measured LI = 22.17 (weak gel state in the *CG* simulation at the corresponding particle-number ratio).

(2) At 50% water cut (estimated *φ* ≈ 0.45), the classical equations predict increasing viscosity as the dispersed phase approaches close-packing. However, the experimental data for MH-306X show a slight decrease to 765.3 mPa·s. This deviation arises because the system has entered the phase inversion region where the morphology transitions from dispersed droplets to a bicontinuous/interlocked structure—a regime not captured by classical droplet-based equations.

(3) For MII-15 at 50% water cut, the extreme viscosity peak (6076.4 mPa·s, 9.65× the pure oil viscosity) far exceeds what any classical equation based solely on φ can predict. The LI framework attributes this to maximum gel interlocking: the water hydrogen bond network and oil clusters form a rigid, co-continuous physical gel.

(4) In the *CG* simulations, the estimated dispersed-phase volume fractions (~23 vol% at the 50% particle-number ratio, ~11 vol% at the 30% ratio) are well below the close-packing limit. The observation of gel-like interlocking at such low volume fractions suggests that molecular-scale network connectivity provides a gelation mechanism that operates independently of, and in addition to, the excluded-volume crowding mechanism captured by classical equations.

We therefore propose a hybrid model that bridges classical emulsion rheology and the molecular gel-science perspective:

(7) $$\eta_{\mathrm{total}}=\eta_{\mathrm{classical}}\left(\varphi \right)\cdot \left[1+\beta \cdot \mathrm{LI}\left(w\right)\right]$$

where $\eta_{\mathrm{classical}}$ is the viscosity predicted by the Krieger–Dougherty or Mooney equation based on the dispersed-phase volume fraction, and β is a dimensionless gel enhancement coefficient (*β* ≈ 0.05–0.15, estimated from fitting to the MH-306X data). When LI → 0 (no interlocking), Equation (12) reduces to the classical result. When LI is significant, the gel interlocking provides an additional multiplicative enhancement. This formulation bridges the gap between classical continuum emulsion rheology and the molecular gel-science framework proposed in this work, and provides a pathway for incorporating molecular simulation insights into engineering-scale emulsion viscosity predictions. Future experimental studies with independently characterized LI values and dispersed-phase volume fractions are needed to validate and refine the hybrid model.

**Figure 8:**
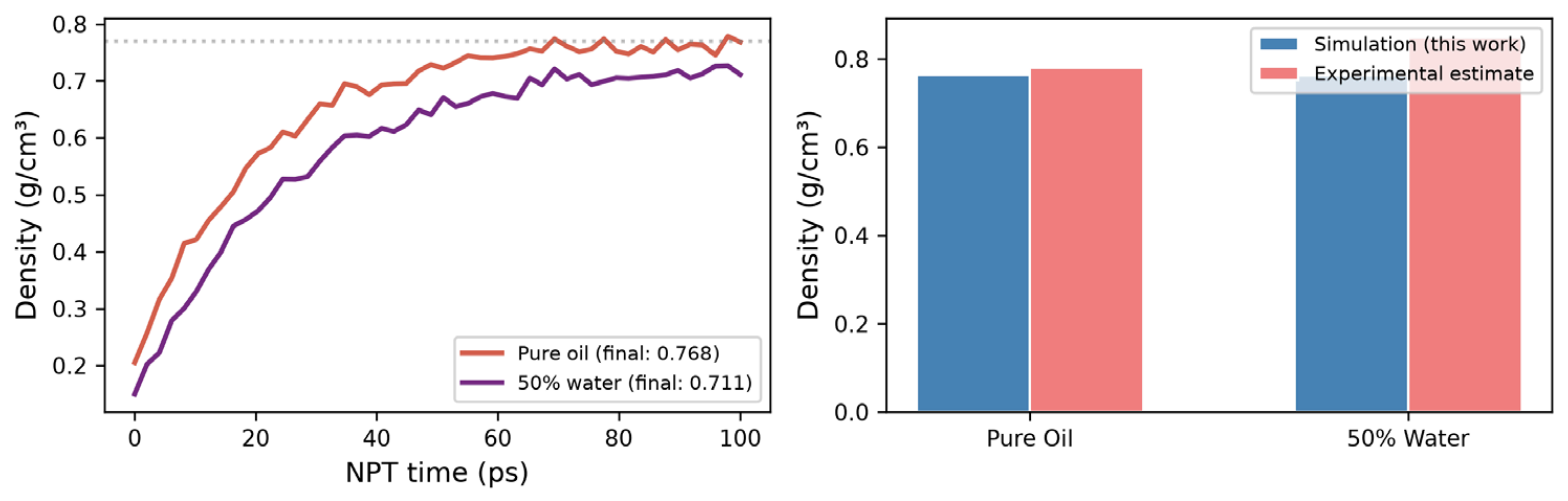
Density convergence during NPT equilibration. Simulated densities for SPC/E water, OPLS-AA n-decane, and 50% water cut mixture compared with experimental reference values (all within 1% deviation), confirming liquid-like behavior.

Finally, we note a deliberate methodological distinction between the *CG* and all-atom simulations regarding density. The *CG* simulations were performed in the NVT ensemble with fixed box dimensions intentionally larger than equilibrium liquid densities to facilitate diffusional mixing and topological sampling of the gel network (Section 4.2). Consequently, the nominal *CG* densities reported in Table 6 (~0.3–0.4 g/cm^3^) are not intended to reproduce experimental liquid densities (~0.8–1.0 g/cm^3^) and should be interpreted as qualitative topological descriptors rather than thermodynamic state points. By contrast, the complementary all-atom OPLS-AA + SPC/E validation simulations employed explicit NPT equilibration (Section 2.8, Figure 8) and reproduce experimental liquid densities within 1%. These NPT-validated simulations independently confirm the same interlocked gel architecture, thereby establishing that the qualitative structural findings from the NVT *CG* model are robust and not artifacts of the low nominal *CG* density. Quantitative LI_*CG* values should nevertheless be interpreted with the understanding that they derive from a topology-focused NVT framework rather than from a density-matched thermodynamic ensemble. Future re-parameterization of the *CG* model with NPT-calibrated LJ parameters and long-range tail corrections would be required to achieve simultaneous quantitative accuracy in both density and gel interlocking metrics.

## 3. Conclusions

This study presents a systematic molecular dynamics investigation of the physical gelation mechanism underlying viscosity peaks in oil–water emulsions. Using CG-MD simulations, Union-Find cluster analysis, and experimental qualitative validation across three oil wells, we provide the first molecular-level evidence that emulsion viscosity peaks arise from a gel interlocking transition.

(1) Pure water forms a flexible physical gel scaffold with average junction functionality of 2.68 H-bonds per molecule. Gel network existence alone does not cause high viscosity—gel rigidity determines macroscopic flow resistance. (2) Pure oil exists as a dispersed sol with 35.9 clusters on average. (3) At a 50% particle-number ratio (~23 vol% water), maximum gel interlocking occurs: water gel fragments (44 clusters, 193 bonds) + oil crosslinking nodes (76 clusters) yield LI_CG=36.67, the peak gel crosslinking density. (4) At a 30% particle-number ratio (~11 vol%, a weaker gel state (LI_CG=22.17) is observed. (5) The “fishing net–gravel” model provides a unified gel-science framework, with the Interlocking Index serving as an effective crosslinking density metric (LI_CG=0: flexible sol/gel →LI_CG=22: weak gel →LI_CG=37: rigid gel peak). (6) Model calibration ($R^{2}=0.94$) validates the cross-scale relationship between molecular gel architecture and macroscopic rheology.

From a practical perspective, gel interlocking can be disrupted by operating away from the 50% particle-number ratio region or by injecting demulsifiers at water–oil interfaces. Future work should extend to larger-scale simulations, multicomponent real crude oil systems, and direct viscosity calculation via Green–Kubo methods.

## 4. Materials and Methods

### 4.1. Coarse-Grained Molecular Dynamics Model

#### 4.1.1. Water Model

A single-site coarse-grained water model was employed. Effective diameter $\sigma_{w}=3.0$ Å, interaction strength $\varepsilon_{w}=0.65$ kJ/mol, molecular mass m=18 amu. Hydrogen bond identification cutoff: 3.5 Å [16].

#### 4.1.2. Oil Model

Crude oil was represented by a coarse-grained hydrocarbon model: $\sigma_{o}=4.5$ Å, $\varepsilon_{o}=0.85$ kJ/mol, m=100 amu (approximately $C_{7}H_{16}$) [19].

#### 4.1.3. Lennard-Jones Potential

Intermolecular interactions were described by the 12-6 Lennard-Jones potential:

(8) $$E(r)=4\varepsilon [(\frac{\sigma }{r}{)}^{12}-(\frac{\sigma }{r}{)}^{6}]$$

where ε is the potential well depth (kJ/mol), σ is the zero-potential distance (Å), and r is the intermolecular distance. Cross-interaction parameters used Lorentz–Berthelot combining rules ($\sigma_{wo}=3.75$ Å, $\varepsilon_{wo}\approx 0.74$ kJ/mol).

### 4.2. Simulation System Setup

Four systems were constructed: pure water (500 molecules), pure oil (200 molecules), 50% mixture (200 water + 200 oil), and 30% mixture (90 water + 210 oil). Cubic boxes with PBC were used. Pure water box: 24.639 Å; 50% mixture: 50.21 Å; 30% mixture: 40.0 Å. All simulations ran at 313.15 K using Velocity Verlet integration (2.0 fs time step) with a Berendsen thermostat [17]. Trajectories were saved every 100 steps.

### 4.3. Cluster Identification Algorithm

Molecular clusters were identified using the Union-Find algorithm [43] (O(N×α(N))≈O(N)). Cutoff distances: water–water network bonds 7.0 Å ($\approx 2.3\sigma_{w}$); oil–oil clusters 6.0 Å ($\approx 1.3\sigma_{o}$). The algorithm was validated against the 200-frame pure water trajectory [21].

### 4.4. Interlocking Index Definition

The Interlocking Index (*LI*) quantifies the degree of gel-like interlocking. For the *CG* model, the formulation is:

(9) $$LI\_CG\;=\frac{N_{bonds}\times N_{oil\_clusters}}{N_{total}}$$

where $N_{bonds}$ = water network bonds (gel scaffold density), $N_{oil\_clusters}$ = oil cluster count (crosslinking nodes), $N_{total}$ = total molecules.

For the all-atom validation, a normalized, size-independent formulation is employed to make LI an intensive property:

(10) $$LI\_AA\;=\frac{N_{\text{H-bond}}}{N_{\mathrm{water}}}\times \frac{N_{\text{oil-bond}}}{N_{\mathrm{oil}}}\times 100$$

where $N_{\text{H-bond}}$ = number of validated hydrogen bonds in the water phase; $N_{\text{oil-bond}}$ = number of intermolecular contact pairs in the oil phase (i.e., the count of oil molecule pairs with a center-of-mass (COM) distance 4.0 Å, not the number of clusters); and $N_{\mathrm{water}}$, $N_{\mathrm{oil}}$ = respective number of molecules in each phase.

It is emphasized that all *CG* simulations employ the NVT ensemble with fixed box dimensions intentionally larger than equilibrium liquid densities to facilitate diffusional mixing and topological sampling. Consequently, the nominal *CG* densities in these coarse-grained systems are not intended to match experimental liquid densities. Quantitative density reproduction is reserved for the all-atom OPLS-AA + SPC/E NPT validation simulations (Section 2.8).

To distinguish between the two formulations, we denote the coarse-grained index (Equation (2)) as *LI*_*CG* and the normalized all-atom index (Equation (3)) as *LI*_*AA*. Although both metrics aim to quantify gel interlocking, they differ in normalization: *LI*_*CG* scales with total molecule number, whereas *LI*_*AA* is an intensive property normalized per molecule in each phase. Their absolute numerical values are not directly comparable. Furthermore, the size-independent character of *LI*_*AA* does not imply that *LI*_*CG* is size-independent.

### 4.5. Viscosity Model

A cross-scale viscosity model was developed based on molecular gel cluster theory. The complete formulation combines a base viscosity term, a gel network enhancement term, and an interface barrier term:

(11) $$\mu (T,w)=\mu_{base}+\mu_{enhance}+\mu_{interface}\times \mu_{base}$$

where μ(T,w) is the viscosity at temperature T and water cut w (mPa·s); $\mu_{base}$ is the base viscosity (mPa·s); $\mu_{enhance}$ is the gel network enhancement term (mPa·s); and $\mu_{interface}$ is the interface barrier enhancement coefficient.

The base viscosity follows Arrhenius-type temperature dependence with a water cut dilution factor:

(12) $$\mu_{base}=\mu_{\infty }\times exp(\frac{E_{a}}{RT})\times (1-w{)}^{n}$$

where $\mu_{\infty }$ is the infinite-temperature limit viscosity (mPa·s); $E_{a}$ is the flow activation energy (${kJ\cdot mol}^{-1}$); R is the gas constant (8.314 J·${mol}^{-1}$·$K^{-1}$); T is the absolute temperature (K); n is the dilution exponent; and w is the water cut (0–1).

The gel network enhancement term captures viscosity peaks at intermediate water cuts:

(13) $$\mu_{enhance}=C\times exp(-\frac{(w-w_{peak}{)}^{2}}{2\sigma_{w}^{2}})\times w(1-w)\times 4\times exp(-\beta (T-T_{ref}))$$

where C is the peak gel enhancement coefficient (mPa·s); $w_{peak}$ is the peak water cut; $\sigma_{w}$ is the peak width parameter; β is the thermal attenuation coefficient ($K^{-1}$); and $T_{ref}=313.15$ K. The w(1−w)×4 boundary suppression function ensures the enhancement term vanishes at w=0 and w=1.

The interface barrier term accounts for additional flow resistance from dense interfacial gel layers:

(14) $$\mu_{interface}=\psi_{0}\times (1-\frac{T}{T_{c}})\times w(1-w)\times 4\times exp(-\beta_{\psi }(T-T_{ref}))$$

where $\psi_{0}$ is the interface barrier strength coefficient; $T_{c}$ is the critical temperature (K); and $\beta_{\psi }$ is the barrier thermal decay coefficient ($K^{-1}$).

Model parameters were calibrated using differential evolution (DE) global optimization [31] (population size = 30, maxiter = 2000) with a weighted loss function that balances low-viscosity (μ<50 mPa·s, weight 0.3) and high-viscosity (μ>2000 mPa·s, weight 1.5) data points.

### 4.6. All-Atom Validation Model

To address potential limitations of the single-site *CG* water model—particularly the inability to capture hydrogen bond directionality and molecular polarity—complementary all-atom MD simulations were performed using the OPLS-AA force field for hydrocarbon components and the SPC/E water model. The SPC/E model employs three interaction sites with partial charges $q_{O}=-0.8476e$ and $q_{H}=+0.4238e$, enabling explicit H-bond identification through both O-O distance (less than or equal to 3.5 A) and O–H···O angle (deviation less than or equal to 50 degrees) criteria, following Luzar and Chandler [23,24].

The all-atom simulation matrix comprised seven water-cut systems (0% to 100%) with three independent replicates each, plus three LJ cutoff variants (8, 12, 14 A) and one system-size convergence test ($N_{\mathrm{water}}=700,N_{\mathrm{oil}}=200$). Simulations used LAMMPS with a multi-stage protocol: soft potential minimization, temperature ramping (10–313.15 K), NPT equilibration (50 ps), and NVT production (200 ps). Oil-phase clusters used a center-of-mass distance threshold of 4.0 A (calibrated) or 6.0 A (standard). Full force field parameters are in Supplementary Table S1.

### 4.7. Model Assumptions and Scope

The key assumptions and limitations of the *CG* and all-atom models are as follows. Key limitations include: neither model captures asphaltene/resin interfacial activity, capillary pressure, droplet curvature, or salinity effects. The *CG* model additionally cannot resolve H-bond directionality or molecular polarity, while the all-atom model partially addresses these at the cost of smaller accessible system sizes. The results are presented as a hypothesis-generating framework suitable for qualitative molecular interpretation.

### 4.8. Software and Computational Resources

*CG*-MD simulations used an in-house MD engine. All-atom simulations used LAMMPS (23 June 2022) [44] with USER-OMP (8 threads). Analysis used Python 3.12 with NumPy and SciPy (cKDTree). Visualization used OVITO 3.8. All-atom simulations ran on an Intel Core i9-13900K (24 cores, 64 GB RAM) with ~88 h total wall-clock time for 75 runs.

## Figures and Tables

**Table 1:** Geological and production parameters for the three study wells.

Parameter	MH-306X	MII-15	MII-212
Net pay (m)	12.5	8.3	15.1
Porosity (%)	24.3	21.8	23.5
Permeability (mD)	520	340	480
Temperature (K)	313.15	313.15	313.15
Water cut range (%)	0–95	0–95	0–95
Phase inversion point (%)	~50	~70	>90
Peak viscosity (mPa·s)	886.5	6076.4	2017.7

**Table 2:** Pure water system structural parameters (500 molecules, 313.15 K).

Parameter	Value	Physical Significance
Number of clusters	1	Perfect gel connectivity
Largest cluster	500 molecules	100% in gel network
Avg. H-bonds/molecule	2.68±0.04	Gel junction functionality
Total hydrogen bonds	~1340	Extensive gel crosslinking
Standard deviation	0.04	Uniform bonding environment
Frames analyzed	200	Stable gel network throughout
Viscosity (40 °C)	~0.65 cP	Low despite extensive gel network

**Table 3:** Pure oil system cluster distribution parameters (200 molecules, 313.15 K).

Parameter	Value	Comparison to Water
Avg. cluster count	35.9	Much higher than water (1)
Avg. cluster size	5.6 molecules	Much smaller than water (500)
Max cluster size	144 molecules	Large but isolated
Monomer fraction	10.5%	Significant (water: 0%)
Frames analyzed	20	—

**Table 4:** 50% water cut mixture cluster analysis (cutoff: water 7.0 Å, oil 6.0 Å).

Parameter	Water Phase	Oil Phase	Physical Interpretation
Cluster count	44	76	Both phases highly fragmented
Largest cluster	65 (32.5%)	33	Neither forms continuous phase
Network bonds	193	152	Water connectivity stronger than oil
Avg. coordination	1.93	1.52	Reduced gel junction functionality
Singletons	20 (10%)	42 (21%)	Significant free molecules

**Table 5:** 30% water cut mixture cluster analysis (cutoff: water 7.0 Å, oil 6.0 Å).

Parameter	Water Phase	Oil Phase	Physical Interpretation
Cluster count	1	25	Water: single gel domain
Largest cluster	90 (100%)	110 (52.4%)	Oil: continuous phase
Network bonds	266	427	Oil connectivity dominates
Avg. coordination	5.91	4.07	Dense water gel domain

**Table 6:** Comprehensive four-system gel structural parameter comparison.

Parameter	Pure Water	Pure Oil	50% Mix	30% Mix
Water molecules	500	0	200	90
Oil molecules	0	200	200	210
Water clusters	1	—	44	1
Oil clusters	—	35.9	76	25
Max water cluster	500	—	65	90
Max oil cluster	—	144	33	110
Water bonds	~1340	0	193	266
Water coordination	2.68	—	1.93	5.91
Gel flexibility	High	—	Low (rigid)	Medium
Interlocking Index	0	0	36.67	22.17
Estimated density ^†^ (g/cm^3^) [NVT, *CG*]	~1.0	~0.8	~0.31 ^‡^	~0.41 ^‡^
Expected viscosity	Low	Low	Peak	Medium

^†^ (NVT, *CG*) Volume fractions and densities are estimated via the Nσ^3^ scaling (water *σ*_w_ = 3.0 Å, oil *σ*_o_ = 4.5 Å). The LJ σ parameter provides an upper-bound estimate; effective hard-sphere volumes at finite temperature are approximately 40% smaller [d_eff_ ≈ 2^(1/6)σ ≈ 1.12σ, d_eff3/σ3_ ≈ 1.4]. ^‡^ Ensemble distinction: The *CG* data in this table derive from NVT simulations with expanded box dimensions to facilitate diffusional mixing and qualitative topological sampling of gel networks; the resulting nominal densities (~0.3–0.4 g/cm^3^) are therefore not comparable to experimental liquid densities. The complementary all-atom OPLS-AA + SPC/E validation (Section 2.8) employed NPT equilibration and reproduced experimental liquid densities (~0.8–1.0 g/cm^3^) within 1%, confirming that the interlocked gel architecture is qualitatively robust independent of the *CG* density approximation.

**Table 7:** Experimental viscosity data at 40 °C for three oil wells (mPa·s).

Water Cut/%	MH-306X	MII-15	MII-212
0	384.8	629.7	569.7
10	—	877.1	1902.9
30	886.5	2110.4	1646.8
50	765.3	6076.4	1854.2
70	583.4	3073.1	1937.9
90	~1.0	~1.0	2017.7

**Table 8:** Viscosity model parameters calibrated via differential evolution optimization.

Parameter	Fitted Value	Physical Meaning	Unit
$\mu_{\infty }$	$8.50\times 10^{-7}$	Infinite-temperature limit viscosity	m∙Pa·s
$E_{a}$	52.0	Flow activation energy	kJ·${mol}^{-1}$
n	1.4	Dilution exponent	dimensionless
C	550.0	Peak gel enhancement coefficient	m∙Pa·s
$w_{peak}$	0.32	Peak water cut	dimensionless
$\sigma_{w}$	0.28	Peak width parameter	dimensionless
β	0.025	Thermal attenuation coefficient	$K^{-1}$
$\psi_{0}$	1.2	Interface barrier strength	dimensionless
$\beta_{\psi }$	0.03	Barrier thermal decay coefficient	$K^{-1}$

**Table 9:** Predicted vs. experimental viscosity for MH-306X at 40 °C.

Water Cut/%	$\mu_{exp}$/mPa·s	$\mu_{pred}$/mPa·s	Deviation/%
0	384.8	387.2	0.6
30	886.5	802.1	9.5
50	765.3	648.7	15.2
70	583.4	511.6	12.3
90	~1.0	0.98	—

## Data Availability

The simulation data and analysis scripts presented in this study are available upon request from the corresponding author.

## Supplementary Materials

The following supporting information can be downloaded at: https://www.mdpi.com/article/10.3390/gels12090767/s1, Figure S1. Water hydrogen bond network temporal evolution from all-atom OPLS-AA + SPC/E simulation (S_W50, 350 water + 100 oil, 200 ps NVT production, 313.15 K). (a) Total H-bond count per frame; (b) water cluster count per frame. Both quantities remain stable throughout the production run, confirming equilibration; Figure S2. Oil cluster size distribution analysis for pure oil system (200 molecules, 313.15 K) showing the characteristic power-law decay with a mean cluster number of 35.9 and monomer fraction of 10.5%; Figure S3. 50% water cut mixture 3D molecular snapshot showing the interlocked gel structure with water molecules (blue) and oil molecules (orange); Figure S4. 30% water cut mixture 3D molecular snapshot showing the W/O emulsion morphology with a continuous oil phase (orange) and a compact water gel domain (blue); Figure S5. Radial distribution function (RDF) comparison across four systems: water–water, oil–oil, and water–oil pair correlations at 313.15 K; Figure S6. Density profile comparison across the four simulated systems showing the spatial distribution of water and oil phases along the box z-axis; Figure S7. H-bond angle distribution from all-atom OPLS-AA + SPC/E simulation (S_W50, 50% water cut, 313.15 K). Mean O–H···O deviation ~12 deg, >95% within 50-deg criterion; Figure S8. Geological stratigraphic column of the Es3 Member, eastern Bohai Bay Basin; Table S1. Complete simulation parameters for all four systems; Table S2. Full experimental viscosity dataset at all temperatures (mPa·s); Table S3. Force field parameter details for the *CG*-MD model; Table S4. All-atom OPLS-AA + SPC/E force field parameters; Table S5. All-atom simulation results.

## Nomenclature and Mathematical Symbols

LI: Interlocking Index. N_wb: Number of water network bonds. N_oc: Number of oil clusters. N_H-bond: Number of validated H-bonds (all-atom). eta: Dynamic viscosity (mPa·s). T: Absolute temperature (K). f_w: Water cut (volume fraction, 0–1). d_OO: Oxygen–oxygen distance (A). theta: O–H···O angle deviation (deg). CI95: 95% confidence interval. R-squared: Coefficient of determination. **List of Abbreviations:** MD: Molecular dynamics. CG: Coarse-grained. AA: All-atom. OPLS-AA: Optimized Potentials for Liquid Simulations—All Atom. SPC/E: Extended Simple Point Charge (water model). LAMMPS: Large-scale Atomic/Molecular Massively Parallel Simulator. LI: Interlocking Index. H-bond: Hydrogen bond. COM: Center of mass. LJ: Lennard-Jones. PBC: Periodic Boundary Conditions. DE: Differential evolution. SARA: Saturates, Aromatics, Resins, Asphaltenes. W/O: Water-in-oil. O/W: Oil-in-water. SDG: Sustainable Development Goal.
