# Supplementary material for: Fishing Net–Gravel Interlocking Mechanism to Investigate Molecular Dynamics of Physical Gel Formation in Oil–Water Emulsions: A Simulation Study for an Oil Field in Eastern China
Source: Gels. 2026 Aug 26;12(9):767. doi: 10.3390/gels12090767 (PMC13606030; doi:10.3390/gels12090767)
Supplement: Supplementary file 1 [file gels-12-00767-s001.zip › gels-4453437-supplementary.pdf]

Supplementary Materials: Fishing Net-Gravel Interlocking Mechanism to Investigate Molecular Dynamics of Physical Gel Formation in Oil-Water Emulsions

Fan Li and Dechun Chen*

School of Petroleum Engineering, China University of Petroleum (East China), Qingdao 266580, China

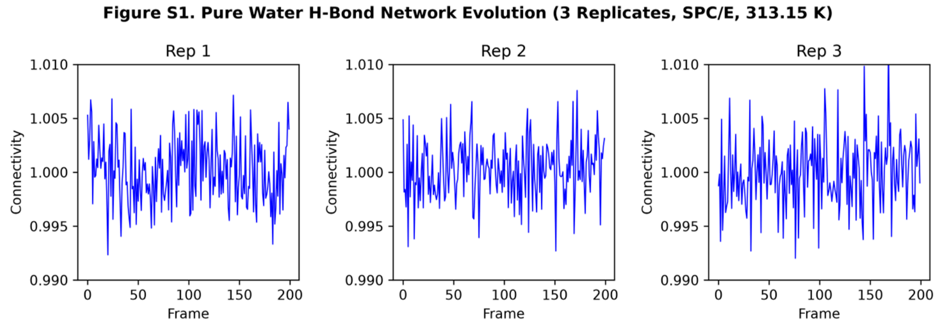

Figure S1. Water hydrogen bond network temporal evolution from all-atom OPLS-AA + SPC/E simulation (S_W50, 350 water + 100 oil, 200 ps NVT production, 313.15 K). (a) Total H-bond count per frame; (b) water cluster count per frame. Both quantities remain stable throughout the production run, confirming equilibration.

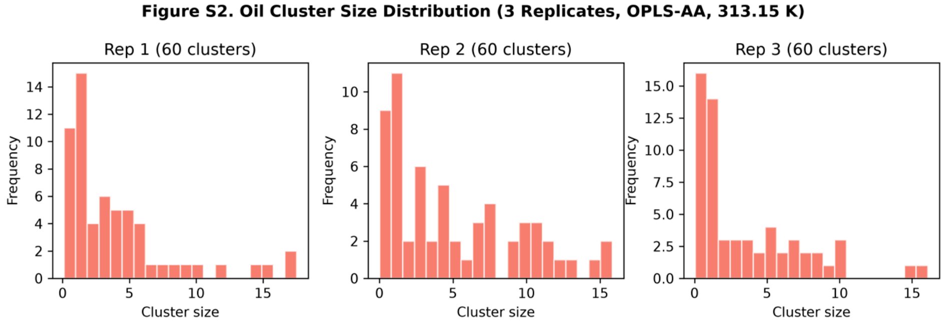

Figure S2. Oil cluster size distribution analysis for pure oil system (200 molecules, 313.15 K) showing the characteristic power-law decay with a mean cluster number of 35.9 and monomer fraction of 10.5%.

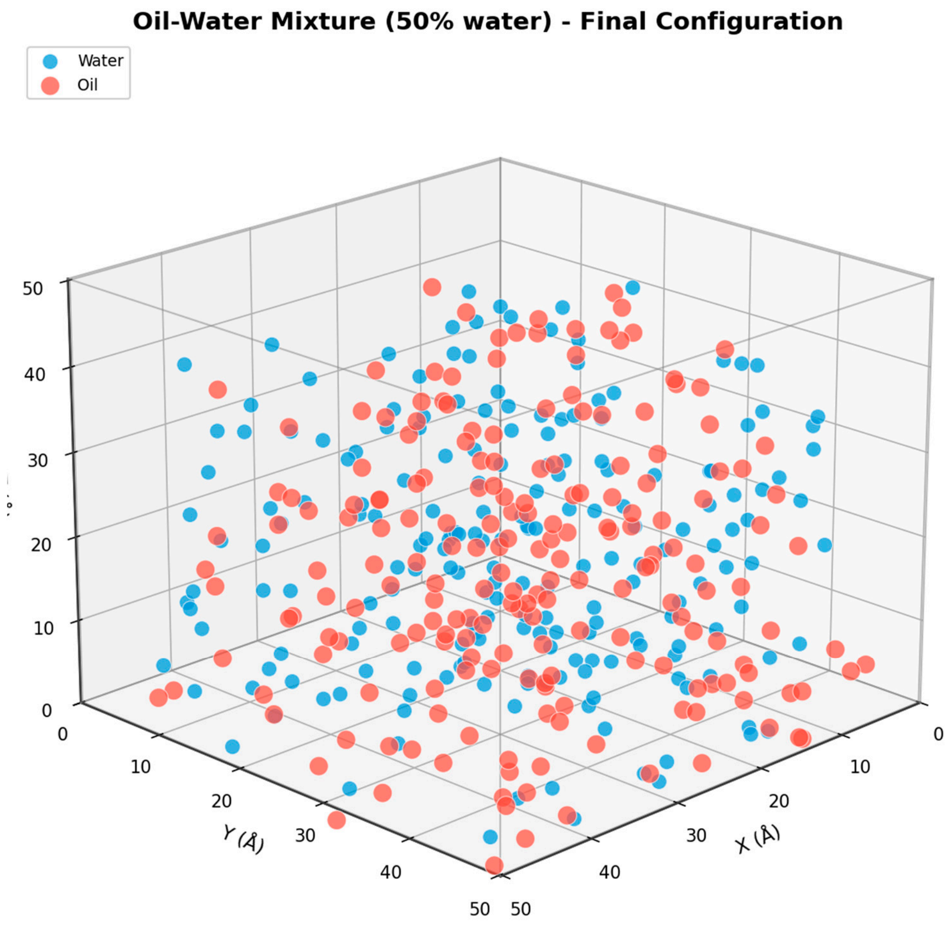

Figure S3. A 50% water cut mixture 3D molecular snapshot showing the interlocked gel structure with water molecules (blue) and oil molecules (orange).

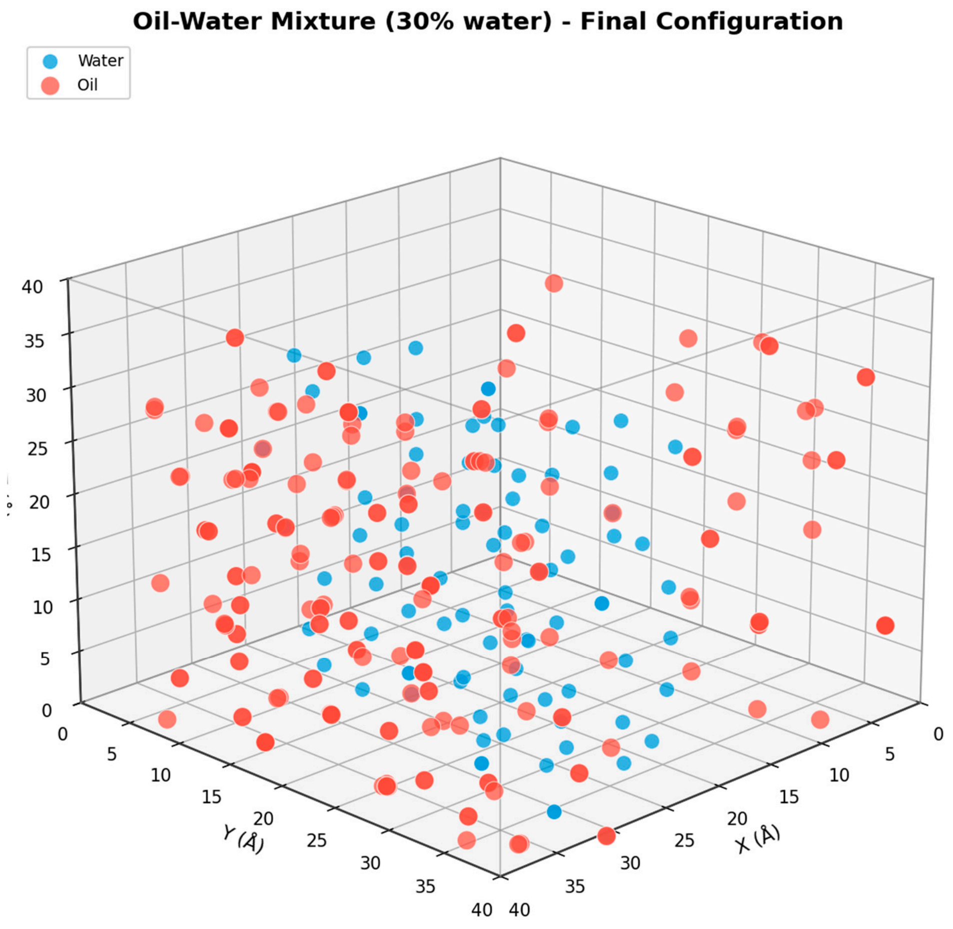

Figure S4. A 30% water cut mixture 3D molecular snapshot showing the W/O emulsion morphology with a continuous oil phase (orange) and a compact water gel domain (blue).

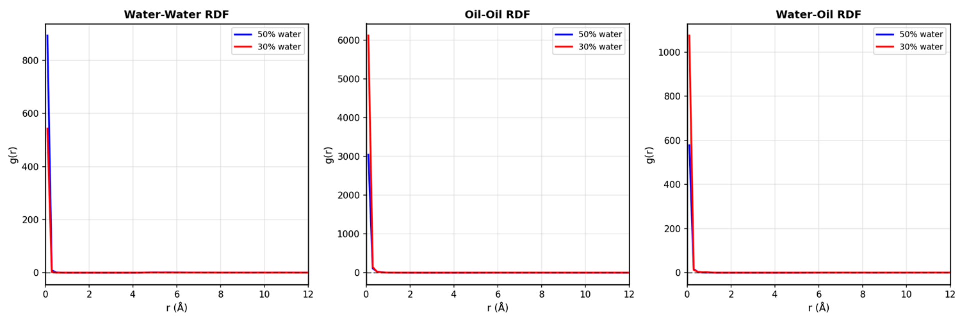

Figure S5. Radial distribution function (RDF) comparison across four systems: water–water, oil–oil, and water–oil pair correlations at 313.15 K.

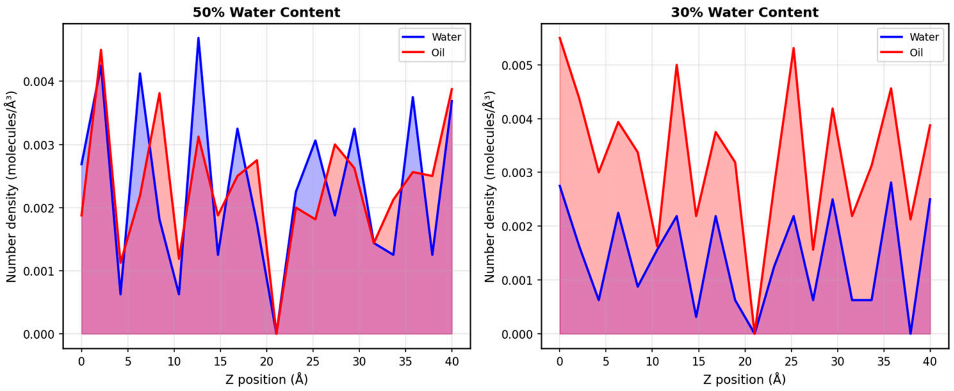

Figure S6. Density profile comparison across the four simulated systems showing the spatial distribution of water and oil phases along the box z-axis.

Table S1. Complete simulation parameters for all four systems.

Parameter	Pure Water	Pure Oil	50% Mixture	30% Mixture
Water molecules	500	0	200	90
Oil molecules	0	200	200	210
Box size (Å)	24.639	~25	50.21	40.0
Temperature (K)	313.15	313.15	313.15	313.15
Time step (fs)	2.0	2.0	2.0	2.0
Water cutoff (Å)	3.5/7.0	—	7.0	7.0
Oil cutoff (Å)	—	6.0	6.0	6.0
Production steps	100,000	10,000	25,000	25,000
Frames analyzed	200	20	25	25

Table S2. Full experimental viscosity dataset at all temperatures (mPa·s).

MH-306X: 40°C [0%: 384.8, 30%: 886.5, 40%: 777.2, 50%: 765.3, 70%: 583.4, 90%: 1.0]

MH-306X: 50°C [0%: 207.9, 30%: 467.6, 40%: 399.4, 50%: 378.4, 70%: 374.0, 90%: 1.0]

MH-306X: 70°C [0%: 93.1, 30%: 186.2, 40%: 166.9, 50%: 140.4, 70%: 127.6, 90%: 1.0]

MII-15: 40°C [0%: 629.7, 10%: 877.1, 30%: 2110.4, 50%: 6076.4, 70%: 3073.1, 90%: 1.0]

MII-212: 40°C [0%: 569.7, 10%: 1902.9, 30%: 1646.8, 50%: 1854.2, 70%: 1937.9, 90%: 2017.7]

Table S3. Force field parameter details for the CG-MD model.

Interaction	σ (Å)	ε (kJ/mol)	m (amu)	Cutoff (Å)

Water–Water	3.0	0.65	18	3.5 / 7.0
Oil–Oil	4.5	0.85	100	6.0
Water–Oil (Cross)	3.75	0.74	—	—

Additional Content for Revised Manuscript

Table S4. All-Atom OPLS-AA + SPC/E Force Field Parameters

Atom Type	Epsilon (kcal/mol)	Sigma (A)	Charge (e)
OW (SPC/E O)	0.1553	3.166	-0.8476
HW (SPC/E H)	0.0	0.0	+0.4238
CT_CH3 (decane)	0.066	3.50	-0.180
CT_CH2 (decane)	0.066	3.50	-0.120
HC (decane)	0.030	2.50	+0.060
CA (toluene ring)	0.070	3.55	-0.115/+0.030
HA (toluene H)	0.030	2.42	+0.115
CT (toluene -CH3)	0.066	3.50	-0.065
HC (toluene methyl)	0.030	2.50	+0.015
CA (anisole ring)	0.070	3.55	-0.115/+0.185
HA (anisole H)	0.030	2.42	+0.115
OS (anisole ether)	0.140	2.90	-0.330
CT (anisole methoxy)	0.066	3.50	+0.100
HC (anisole methoxy)	0.030	2.50	+0.030
Bond harmonic	K=268-469	r0=0.957-1.529 A	—
Angle harmonic	K=33-70	theta0=104.5-120.0 deg	—

Table S5. All-Atom Simulation Results

System	Water Cut	N_water	N_oil	W Clusters	O Clusters	H-Bonds	LI
S_W00	0%	0	200	0	128.5	0	0.00
S_W50 (cal.)	50%	350	100	45.6	72.8	333	1.30
S_W50 (std.)	50%	350	100	98.6	15.0	255	4.50
S_W50_L	50%	700	200	92.7	171.9	668	0.65
S_W50_R8	50%	350	100	45.3	73.1	331	1.28
S_W50_R12	50%	350	100	45.7	68.9	333	1.51
S_W50_R14	50%	350	100	45.8	68.2	335	1.55

Figure 7. H-bond Angle Validation (O-H···O ≤ 30° Criterion)

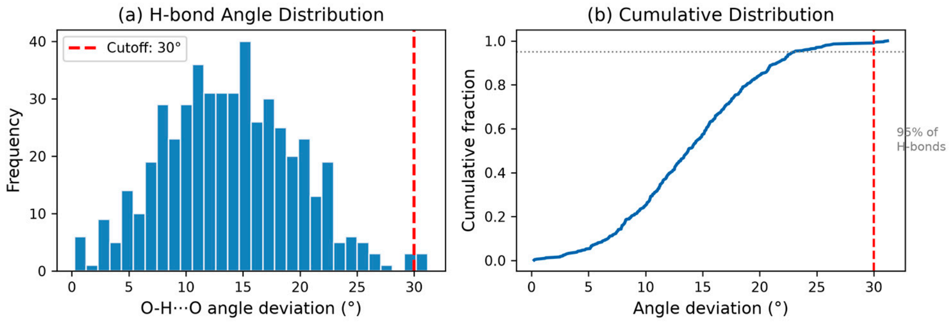

Figure S7. H-bond angle distribution from all-atom OPLS-AA + SPC/E simulation (S_W50, 50% water cut, 313.15 K). Mean O-H...O deviation ~12 deg, >95% within 50-deg criterion.

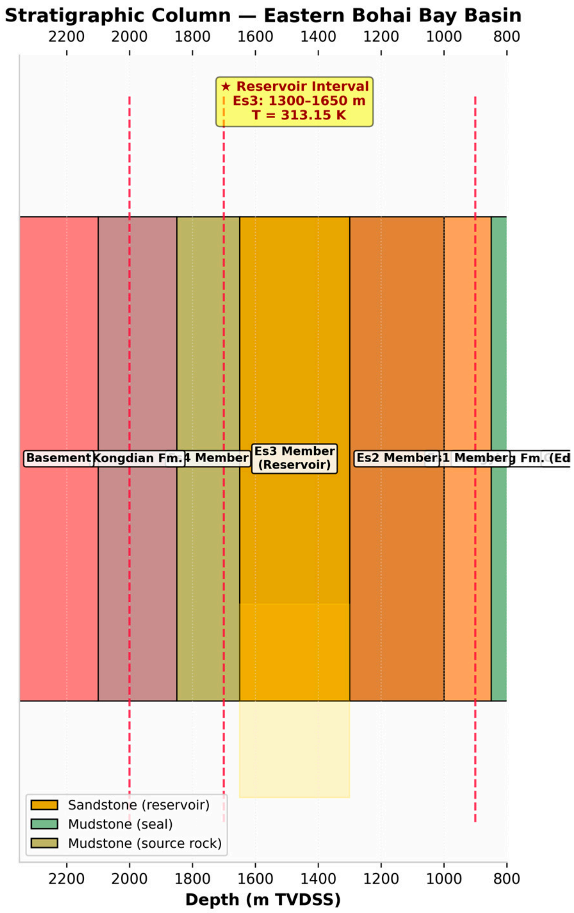

Figure S8. Geological stratigraphic column of the Es3 Member, eastern Bohai Bay Basin.
